# Curing Parthenogenesis-Inducing (PI) *Wolbachia*-Induced Reproductive Disorders in the Egg Parasitoid *Telenomus remus*

**DOI:** 10.3390/biology15030210

**Published:** 2026-01-23

**Authors:** I-Cheng Tu, Ching-Ting Lai, Li-Hsin Wu

**Affiliations:** Department of Plant Medicine, National Pingtung University of Science and Technology, Neipu, Pingtung 912, Taiwan; gundam2001101@gmail.com (I.-C.T.); playford7129@gmail.com (C.-T.L.)

**Keywords:** *Wolbachia*, *Telenomus remus*, reproductive isolation, life-cycle analysis

## Abstract

*Wolbachia* is an endosymbiotic bacterium widespread in invertebrates that causes various reproductive effects, including cytoplasmic incompatibility, feminization, male killing, and the induction of parthenogenesis (PI). PI-*Wolbachia w*Rem converts *Telenomus remus*, an egg parasitoid of *Spodoptera frugiperda*, from arrhenotokous reproduction (male-producing) to thelytokous reproduction (female-producing). Long-term symbiosis between egg parasitoids and *Wolbachia* has been shown to lead to reproductive barriers and “female functional virginity,” causing progressive and potentially irreversible sex ratio imbalances. However, whether such reproductive barriers occur in *T. remus* remains unknown, which has important implications for biological control programs utilizing this parasitoid. To address this question, we cured *w*Rem using tetracycline and conducted crossing experiments with naturally uninfected strains (W-). The results indicated that the cured strain (Wcure) retained normal sexual reproductive capability, with self-crossing fertilization rates comparable to those of W- strains. However, first-generation hybridization between Wcure and W- strains produced strongly male-biased offspring (male proportion: 94.3% and 85.8% for W-♂ × Wcure♀ and Wcure♂ × W-♀, respectively), indicating substantial reproductive incompatibility. Notably, an asymmetric pattern was observed between reciprocal crosses. In second-generation hybridization experiments, hybrid females (W-/Wcure) mated with W- or Wcure males showed markedly recovered sex ratios (male proportion: 14.3% and 15.6%, respectively), although total offspring numbers remained lower than in self-crossing groups. These results suggest that the reproductive incompatibility in *T. remus* differs from female functional virginity and is more consistent with mitonuclear incompatibility arising from population divergence. The partial recovery in second-generation hybrids indicates that surviving F1 hybrid females likely represent individuals selected for compatibility, rather than exhibiting progressive deterioration of sexual function. These findings offer insights into *Wolbachia*’s impact on parasitoid reproduction and highlight key considerations for biological control applications, underscoring the importance of evaluating reproductive barriers before deploying cured strains and preventing symbiont loss within populations.

## Simple Summary

Certain bacteria live inside insects and can dramatically alter how they reproduce. One such bacterium, called *Wolbachia*, infects a tiny parasitic wasp (*Telenomus remus*) that naturally controls fall armyworm, a destructive crop pest. This bacterium causes infected female wasps to produce only female offspring without mating, which could be advantageous for pest control programs that rely on female wasps to attack pest eggs. However, previous studies on related wasp species suggested that long-term infection may permanently damage the wasps’ ability to reproduce normally. We removed the bacterium using antibiotics and tested whether cured wasps could still mate and produce offspring normally. While cured wasps reproduced normally among themselves, crosses between cured and naturally uninfected wasps produced almost exclusively male offspring, indicating a breeding barrier between these two populations. Importantly, this barrier partially disappeared in the next generation, suggesting it results from genetic differences accumulated during their separate evolutionary histories rather than permanent reproductive damage. These findings are valuable for agricultural pest management because they reveal that mixing wasp populations with different infection histories may cause unexpected breeding problems, potentially undermining biological control efforts against crop pests.

## 1. Introduction

*Wolbachia* is a widespread intracellular endosymbiont found in invertebrates, including insects, nematodes, and other arthropods [1,2]. To enhance its transmission within host populations, *Wolbachia* has evolved various physiological and behavioral manipulation strategies that facilitate the establishment and maintenance of stable infections. These reproductive manipulations include cytoplasmic incompatibility (CI), male killing, feminization, and parthenogenesis induction (PI) [3].

PI-*Wolbachia* primarily affects insects with haplodiploid sex determination systems, in which unfertilized haploid eggs develop into males while fertilized diploid eggs develop into females. Typically, these insects reproduce sexually, and unmated females produce only male offspring, resulting in arrhenotokous populations comprising both sexes [4]. However, under PI-*Wolbachia* infection, processes such as mitosis, meiosis, or chromosome duplication may be disrupted [5,6], resulting in incomplete chromosomal segregation in haploid eggs. Consequently, these eggs become diploid and develop into female offspring [4]. This leads to thelytokous parthenogenesis, causing the population sex ratio to become highly female-biased or exclusively female, which facilitates the rapid spread of the symbiont.

Despite its benefits to *Wolbachia* transmission, PI-*Wolbachia* infection can impose substantial costs on the host. The reproductive manipulation and physiological interference are often accompanied by various adverse effects [7]. Studies on *Telenomus nawai* [8] and *Trichogramma pretiosum* [9] demonstrated that when antibiotic-cured females were crossed with males from uninfected strains, increasingly male-biased sex ratios were observed as the proportion of cured-strain genetics increased through successive generations. This pattern suggests that infected females gradually lose their ability to reproduce sexually. Such degeneration of female sexual function, which may become irreversible after several generations, is termed “female functional virginity” [8].

Three main hypotheses have been proposed to explain this loss of sexual reproductive capability [9]: (1) mutation accumulation, where relaxed selection on sexual reproduction alleles under thelytoky allows neutral mutations to accumulate, leading to gradual deterioration of both male and female sexual functions [10]; (2) costly female trait, where nonfunctional but energetically costly female sexual traits are eliminated by natural selection, while male traits deteriorate due to disuse [11]; and (3) virginity mutation, where female-biased sex ratios promote selection for the loss of female sexual function, while male reproductive functions remain under selection and are retained [12].

*Telenomus remus* (Nixon) (Hymenoptera: Scelionidae) is an egg parasitoid native to the Malay Peninsula and Papua New Guinea [13], and has been widely deployed for biological control of fall armyworm (*Spodoptera frugiperda*) in various countries worldwide [14,15,16,17,18,19,20,21,22]. Our team discovered PI-*Wolbachia*-infected populations of this species in Pingtung, southern Taiwan. Given prior reports of female functional virginity in *T. nawai* and *Tr. pretiosum*, it is plausible that *T. remus* might exhibit similar reproductive effects.

From a biological control perspective, the composition of parasitoid populations—particularly the female-to-male ratio—is crucial, as a higher proportion of females generally enhances the capacity to suppress pest populations. Therefore, PI-*Wolbachia*-induced thelytokous reproduction could offer a significant advantage by increasing the number of reproductive females [23]. However, it remains unknown whether reproductive barriers similar to female functional virginity occur in *T. remus*, and whether curing PI-*Wolbachia* would restore normal sexual reproduction or induce incompatibility with naturally uninfected populations. These potential barriers must be evaluated before deploying infected or cured strains in biological control programs.

This study aimed to: (1) determine whether curing PI-*Wolbachia* from *T. remus* restores normal sexual reproduction and (2) assess reproductive compatibility between cured and naturally uninfected strains. We hypothesized that, if female functional virginity occurs in *T. remus*, cured females crossed with uninfected males would show male-biased sex ratios as the proportion of cured-strain genetics gradually increases through successive generations. We also explored the potential mechanisms underlying these effects based on the three hypotheses outlined above. Our findings provide essential information for risk assessment of PI-*Wolbachia*-infected *T. remus* strains and contribute to the development of more efficient pest management strategies in southern Taiwan.

## 2. Materials and Methods

### 2.1. Rearing of Telenomus remus

The *T. remus* strains used in this study, including *Wolbachia*-infected, uninfected, and antibiotic-cured strains, were collected from Wandan, Pingtung, Taiwan. All experiments were conducted in growth chambers at 25 °C, 80 ± 10% relative humidity, and a 12:12 h light–dark photoperiod. Parasitoid wasps were reared in 50 mL centrifuge tubes using fall armyworm (*Spodoptera frugiperda*, Lepidoptera: Noctuidae) eggs as hosts, and adults were fed pure honey [8]. Three strains were established: (1) W+ strain: *Wolbachia*-infected strain; (2) W- strain: naturally *Wolbachia*-uninfected strain, collected from the same locality as the W+ strain and confirmed to be uninfected by PCR screening using *wsp* primers; (3) Wcure strain: *Wolbachia*-cured strain, derived from the W+ strain through antibiotic treatment.

### 2.2. Antibiotic Treatment

**Figure 1:**
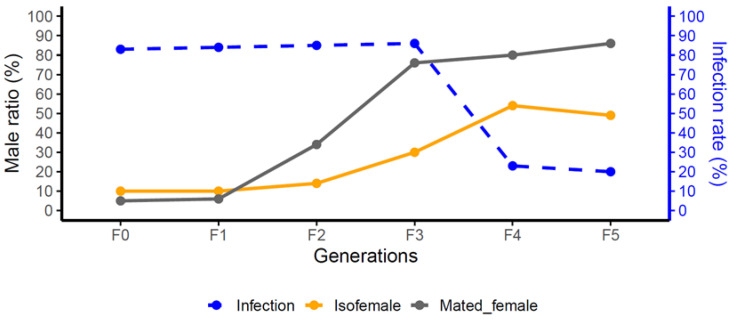
Changes in *Wolbachia* infection rate and male ratio across generations (F0–F5). Blue dashed line indicates infection rate; orange and gray solid lines represent male ratio in isofemale and mated female lines, respectively.

Across fifteen generations, the *Wolbachia*-infected *T. remus* strain was fed honey water containing 0.1% tetracycline for the first ten generations, followed by five generations without tetracycline, during which the male proportion and infection rate were recorded (Figure 1). In each generation, PCR was performed using *wsp* primers (*wsp*81F: 5′-TGGTCCAATAAGTGATGAAGAAAC-3′; *wsp*691R: 5′-AAAAATTAAACGCTACTCCA-3′), and *Wolbachia* infection status was monitored through agarose gel electrophoresis until no *Wolbachia* signal was detected [8].

### 2.3. Construction of Wolbachia Phylogenetic Tree

The phylogenetic position of the *Wolbachia* strain infecting *T. remus*, designated as *w*Rem, was determined using the Maximum Likelihood (ML) method based on the *wsp* gene sequence (*n* = 1).

### 2.4. Crossing Experiments (Genetic Introgression Experiment)

**Figure 2:**
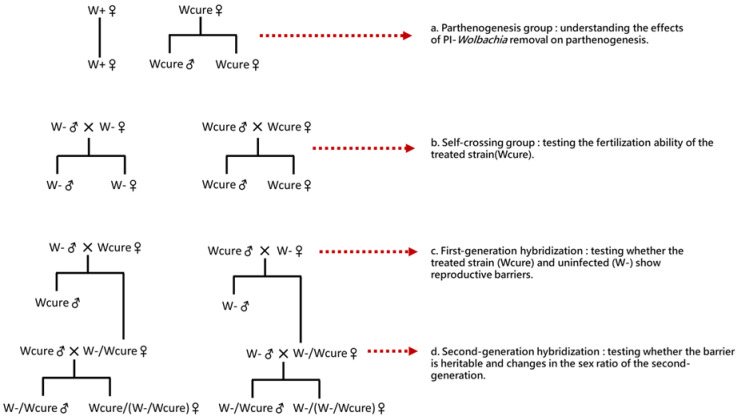
Experimental design for investigating the effects of *Wolbachia* removal on reproduction and hybridization. (**a**) Parthenogenesis group: comparing offspring production between *Wolbachia*-infected (W+) and cured (Wcure) females to understand the effects of PI-*Wolbachia* removal on parthenogenesis; (**b**) Self-crossing group: testing the fertilization ability of the treated strain (Wcure) compared to uninfected controls (W-); (**c**) First-generation hybridization: reciprocal crosses between Wcure and W- strains to test whether reproductive barriers exist between treated and uninfected individuals; (**d**) Second-generation hybridization: crosses using F1 hybrids to examine whether the reproductive barrier is heritable and to assess changes in the sex ratio of the second generation.

Following the methodology of Jeong and Stouthamer [8], the three strains were subjected to parthenogenetic and mating treatments, establishing four experimental groups (Figure 2). Experimental conditions remained consistent with those described above, with a generation cycle of 13 days. Three days post-emergence, adult parasitoids were provided with egg cards containing 100 ± 10 host eggs. The sex ratio, numbers of males and females, and total offspring were recorded after 10 days.

(1) Parthenogenesis Group (Figure 2a)

To assess the effect of *Wolbachia* curing on *T. remus* parthenogenesis, the W+ strain served as a control for comparison with the Wcure strain. Virgin females were isolated individually in 1.5 mL centrifuge tubes for parasitism (W+: *n* = 36; Wcure: *n* = 206).

(2) Self-Crossing Group (Figure 2b)

To confirm the sexual reproductive capability of the Wcure strain, each strain was subjected to intra-strain mating with a male-to-female ratio of 5:1. The naturally uninfected W- strain, which produces female offspring through sexual reproduction, served as a control. After 24 h of mating, females were isolated individually for parasitism (W- ♂ × W- ♀: *n* = 247; Wcure♂ × Wcure♀: *n* = 239).

(3) First-Generation Hybridization (Figure 2c)

Reciprocal hybridization between the W- and Wcure strains was performed to assess whether curing PI-*Wolbachia* induces reproductive incompatibility. Two crosses were established: W- ♂ × Wcure♀ and Wcure♂ × W- ♀, with a male-to-female ratio of 5:1. After 24 h of mating, females were isolated individually for parasitism (W- ♂ × Wcure♀: *n* = 240; Wcure♂ × W- ♀: *n* = 224).

(4) Second-Generation Hybridization (Figure 2d)

To determine whether the reproductive incompatibility induced by curing PI-*Wolbachia* persists in subsequent generations, first-generation hybrid females (W- /Wcure♀) were backcrossed with W- and Wcure males. Two crosses were established: W- ♂ × W- /Wcure♀ and Wcure♂ × W- /Wcure♀, with a male-to-female ratio of 5:1. After 24 h of mating, females were isolated individually for parasitism (W- ♂ × W- /Wcure♀: *n* = 192; Wcure♂ × W- /Wcure♀: *n* = 189).

### 2.5. Statistical Analysis

All analyses were performed in R (version 4.3.2). Count data (numbers of females, males, and total offspring) were analyzed using negative binomial generalized linear models (GLMs), and male proportion data were analyzed using quasi-binomial GLMs to account for overdispersion. Treatment effects were tested using Type II likelihood ratio tests, followed by pairwise comparisons with Tukey’s adjustment (α = 0.05).

### 2.6. Use of Generative AI

Generative AI (Claude 4.5, Anthropic, San Francisco, CA, USA, 2025) was used to assist with language editing, including grammar, clarity, and structure improvements of the manuscript. All AI-generated suggestions were critically reviewed, verified, and edited by the authors, who take full responsibility for the final content.

## 3. Results

### 3.1. Antibiotic Treatment

As shown in Figure 1, successive generations of *T. remus* treated with tetracycline exhibited a gradual decline in *Wolbachia* infection rate (blue line). In contrast, the male proportion among offspring of both virgin females (orange line) and mated females (gray line) increased significantly. These results indicate that curing PI-*Wolbachia* can alter the reproductive pattern of *T. remus*. Furthermore, electrophoresis results (Figure S1) confirmed that *Wolbachia* signals were undetectable for five consecutive generations after treatment, indicating that the *Wolbachia* had been completely eradicated from the infected strain. Therefore, the Wcure strain used in this study is free from any potential confounding effects of *Wolbachia*.

### 3.2. Construction of Wolbachia Phylogenetic Tree

**Figure 3:**
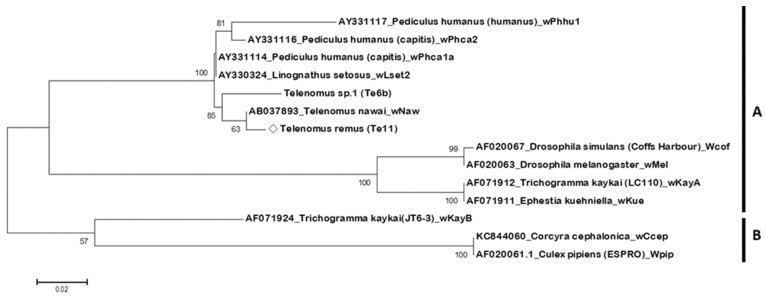
Phylogenetic tree of *Wolbachia* based on *wsp* gene sequences, constructed using the Maximum Likelihood method. Bootstrap values from 1000 replicates are shown on branches; values below 50% are not displayed. Scale bar indicates nucleotide substitutions per site. The diamond symbol (◇) indicates the *Wolbachia* strain identified in this study. The *Wolbachia* strain *w*Rem (detected in *Telenomus remus* individual Te11) forms a monophyletic group in clade A with *w*Naw from *T. nawai* and an unconfirmed strain Te6b. Clade B includes strains from various insect hosts, such as *Trichogramma kaykai*, *Corcyra cephalonica*, and *Culex pipiens*.

As shown in Figure 3, the symbiotic *Wolbachia* strain *w*Rem (detected in *T. remus* individual Te11) forms a monophyletic group in clade A with the *Wolbachia* strain *w*Naw, previously reported in *T. nawai*, and an unconfirmed strain Te6b.

### 3.3. Crossing Experiments

(1) Parthenogenesis Group

In the parthenogenesis group, the W+ strain exhibited a low male proportion (mean = 0.05702) (Table 1), consistent with an active PI effect and a female-biased sex ratio. In contrast, the Wcure strain showed a significantly higher male proportion under parthenogenesis (mean = 0.9354) (Table 1). GLM post hoc comparison confirmed that the male proportion of Wcure♀ was significantly higher than that of W+♀ (odds ratio = 0.004176, z = −12.06, *p* < 0.001) (Table S4). These results, together with the data in Figure 1, demonstrate that curing *w*Rem disrupts the PI effect.

(2) Self-Crossing Group

To assess whether the Wcure strain retained sexual reproductive capability, self-crosses (Wcure♂ × Wcure♀, Figure 2b) were conducted. The W- strain served as a biologically relevant control, as it is naturally uninfected and produces female offspring through sexual reproduction. Results showed a similar sex ratio between the two strains (GLM post hoc: odds ratio = 0.7495, z = −2.261, *p* = 0.316) (Table S4), indicating that the Wcure strain retained normal sexual reproductive function and could produce female offspring after curing *w*Rem.

(3) Hybridization Group

**Figure 4:**
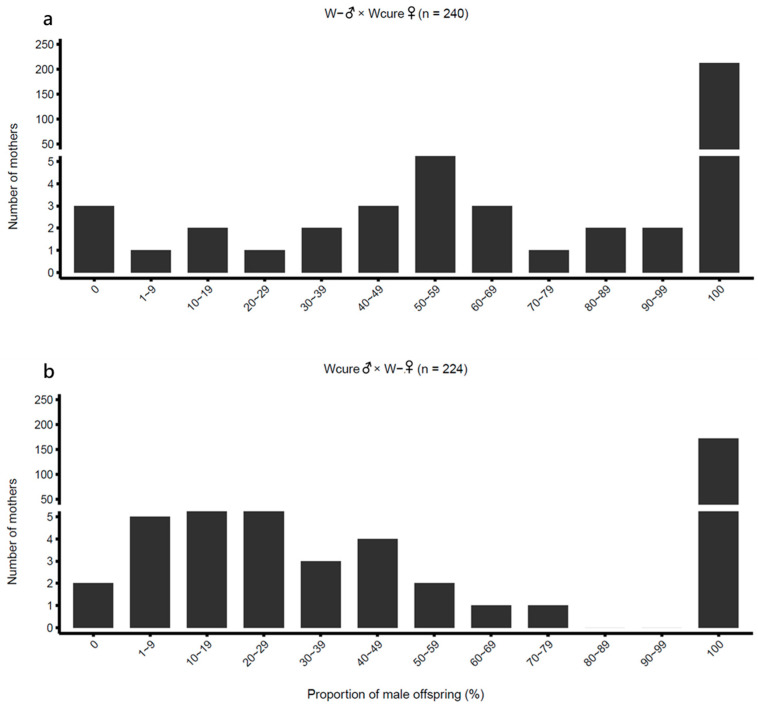
Distribution of male offspring proportion in reciprocal crosses between W- and Wcure strains. (**a**) W-♂ × Wcure♀ (*n* = 240); (**b**) Wcure♂ × W-♀ (*n* = 224). Data were analyzed using quasi-binomial GLMs (*p* < 0.05).

First-generation hybridization crosses between Wcure and W- strains produced abnormally high male proportions (0.9530 and 0.8579 for W- ♂ × Wcure♀ and Wcure♂ × W- ♀, respectively), displaying a strongly male-biased sex ratio (Table 1 and Figure 4). GLM post hoc comparisons confirmed that the male proportions of both hybrid combinations were significantly higher than those of the self-crossing groups (W- ♂ × Wcure♀ vs. W- ♂ × W- ♀: odds ratio = 0.01363, z = −21.75, *p* < 0.001; Wcure♂ × W- ♀ vs. Wcure♂ × Wcure♀: odds ratio = 16.38, z = 20.02, *p* < 0.001) (Table S4). Furthermore, the male proportion of W- ♂ × Wcure♀ was significantly higher than that of Wcure♂ × W- ♀ (odds ratio = 3.356, z = 5.889, *p* < 0.001) (Table S4), revealing an asymmetric pattern between reciprocal crosses. These findings demonstrate that the two strains are nearly incapable of producing female offspring, indicating substantial reproductive incompatibility.

**Figure 5:**
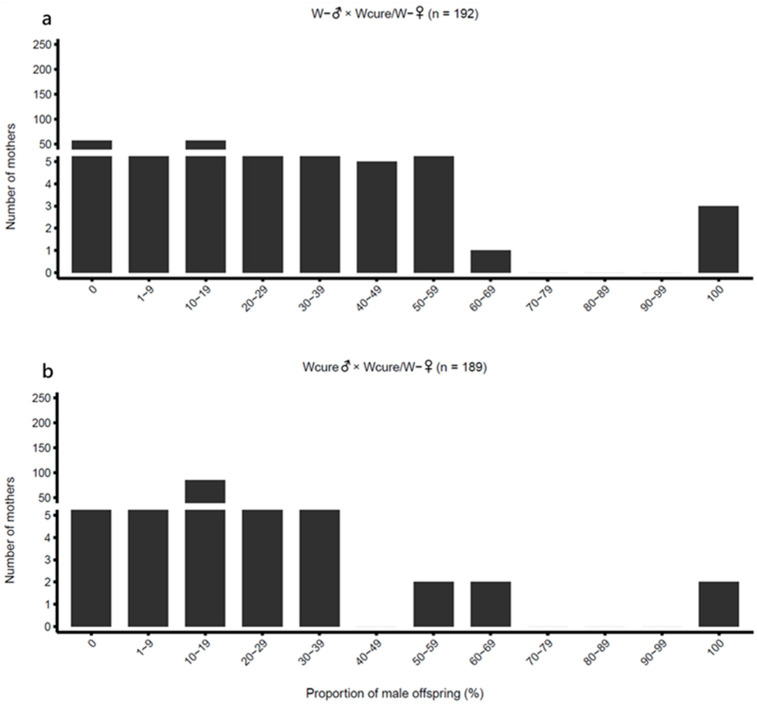
Distribution of male offspring proportion in second-generation hybridization crosses. (**a**) W-♂ × W-/Wcure♀ (*n* = 192); (**b**) Wcure♂ × W-/Wcure♀ (*n* = 189). Data were analyzed using quasi-binomial GLMs (*p* < 0.05).

In second-generation hybridization experiments, the few hybrid females (W- /Wcure) produced in the first generation, when mated with males from their paternal strains (either W- ♂ or Wcure♂), showed a marked reduction in male proportion to 0.1432 and 0.1563, respectively, and regained normal female production (Table 1 and Figure 5). GLM post hoc comparisons confirmed that the male proportions of second-generation hybrids were significantly lower than those of first-generation hybrids (W- ♂ × Wcure♀ vs. W- ♂ × W- /Wcure♀: odds ratio = 0.008244, z = −19.65, *p* < 0.001; Wcure♂ × W- ♀ vs. Wcure♂ × W- /Wcure♀: odds ratio = 32.59, z = 20.33, *p* < 0.001) (Table S4). Additionally, no significant difference in male proportion was detected between the two second-generation hybrid combinations (W- ♂ × W- /Wcure♀ vs. Wcure♂ × W- /Wcure♀: odds ratio = 0.9017, z = −0.4788, *p* < 0.001) (Table S4), in contrast to the asymmetric pattern observed in the first generation. Furthermore, the male proportions of second-generation hybrids showed no significant difference from the W- self-crossing group (W- ♂ × W- /Wcure♀ vs. W- ♂ × W- ♀: odds ratio = 1.654, z = 2.574, *p* = 0.165; Wcure♂ × W- /Wcure♀ vs. W- ♂ × W- ♀: odds ratio = 1.491, z = 2.472, *p* = 0.207) (Table S4), but differed significantly from the Wcure self-crossing group (Wcure♂ × W- /Wcure♀ vs. Wcure♂ × Wcure♀: odds ratio = 0.5027, z = −4.367, *p* < 0.001) (Table S4). These results demonstrate that increased genetic introgression weakened rather than reinforced reproductive incompatibility.

To evaluate whether reproductive capacity was fully restored in F2 hybrids, the total offspring number was compared with the self-crossing groups (W- ♂ × W- ♀ and Wcure♂ × Wcure♀) (Table S1). GLM post hoc comparisons revealed that the W- ♂ × W- /Wcure♀ group produced significantly fewer total offspring than the W- self-crossing group (ratio = 1.878, z = 7.059, *p* < 0.001) (Table S2). In contrast, the Wcure♂ × W- /Wcure♀ group did not differ significantly from the self-crossing groups (ratio = 1.160, z = 1.666, *p* = 0.709) (Table S2). Although male proportions were substantially reduced, the W- ♂ × W- /Wcure♀ group still showed reduced offspring production. To further assess female production capacity, female offspring numbers were compared between F2 hybrids and self-crossing controls. The W- ♂ × W- /Wcure♀ group produced significantly fewer females than the W- self-crossing group (ratio = 1.720, z = 3.260, *p* = 0.0247) (Table S3). In contrast, the Wcure♂ × W- /Wcure♀ group showed no significant difference (ratio = 1.089, z = 0.5138, *p* ≈ 1.0) (Table S3).

Overall, the data distribution of the second-generation hybrids fell between that of the first-generation hybrids and the self-crossing groups, suggesting that the few surviving hybrid females (W- /Wcure) from the first generation may not have exhibited strong reproductive incompatibility, or that such incompatibility was partially overcome. This pattern indicates that the reproductive incompatibility observed in *T. remus* differs from the phenomenon previously reported in *T. nawai*.

## 4. Discussion

Extensive research has been conducted on the effects of *Wolbachia*, a microorganism that is widely distributed among insect species. Through its interactions with host organisms, *Wolbachia* exerts considerable influence over various aspects of host physiology, behavior, and evolutionary processes [24]. In this study, we found that removing PI-*Wolbachia w*Rem from *Telenomus remus* resulted in reproductive incompatibility: females were unable to produce female offspring, even after successful mating. This finding is consistent with the results reported by Jeong and Stouthamer [8] in the egg parasitoid *Telenomus nawai*. However, the second-generation hybridization outcomes differed from those reported previously. We discuss these discrepancies from three perspectives: (1) inter-host differences, (2) *Wolbachia*-induced reproductive isolation, and (3) the effects of symbiont-induced PI mechanisms on the host.

(1) Inter-host Differences

Different reproductive incompatibility outcomes may be related to host differences. Fujii et al. [25] demonstrated that the same *Wolbachia* strain can produce different effects in different hosts—the same strain causes feminization in *Ostrinia scapulalis* but induces male-killing in *Ephestia kuehniella*, indicating that identical or highly similar strains can produce different phenotypic effects depending on the host.

We propose that the *w*Rem strain, which infects *T. remus*, and the *w*Naw strain, which infects *T. nawai*, are phylogenetically related and are both classified within the PI-*Wolbachia* group (Figure 3). However, host differences may lead to different phenotypic effects, thus resulting in the observed differences in second-generation hybridization outcomes.

(2) *Wolbachia*-Induced Reproductive Isolation

The results from the self-crossing group confirmed that males of both strains retained sexual functionality, which had not been compromised by *Wolbachia* symbiosis. As a result, we can confidently dismiss both the mutation accumulation hypothesis and the costly female trait hypothesis. Moreover, the considerable production of male offspring in the first-generation hybridization group indicates a reproductive incompatibility between the two strains, potentially tied to a loss of female sexual function. Consequently, the virginity mutation hypothesis appears to be the most consistent explanation for our findings.

Jeong and Stouthamer [8] proposed the concept of “functional virginity,” in which the introgression of genes from the Wcure strain into the W- strain leads to a gradual increase in the male proportion, ultimately resulting in extreme male bias. A similar reproductive barrier has been reported in *Trichogramma pretiosum* [9], suggesting that such obstacles can occur across different species.

The reproductive barrier observed in this study developed at a faster rate than expected; a significant male bias was immediately apparent after the first hybridization cross. This pattern contrasts with the gradual effects associated with “functional virginity” and more closely aligns with the concept of reproductive isolation. Charlat et al. [24] suggested that cytonuclear coevolution between *Wolbachia* and its host may accelerate the divergence of populations with different infection statuses, thereby promoting reproductive isolation.

We hypothesize that the reproductive barrier observed in the present study may result from mitonuclear incompatibility (i.e., incompatibility between mitochondrial and nuclear genomes), which could disrupt normal embryonic development in diploid female offspring, leading to their death and consequently producing an extreme male-biased sex ratio [26]. This hypothesis is supported by evidence from other *Wolbachia*-infected insect systems. Linkage disequilibrium between *Wolbachia* and mtDNA was first documented in *Drosophila simulans*, where the spread of *Wolbachia* infection resulted in the hitchhiking of associated mitochondrial haplotypes through populations [27,28]. More recently, studies on *Polytremis* butterflies demonstrated that *Wolbachia* infections establish tight associations with specific mtDNA haplotypes, with infected individuals showing significantly reduced mitochondrial diversity compared to uninfected individuals [29]. In PI-*Wolbachia* systems, this linkage is expected to be even stronger, as infected females reproduce parthenogenetically without sexual recombination, potentially leading to tighter co-adaptation among *Wolbachia*, mtDNA, and nuclear genomes over evolutionary time. Such *Wolbachia*-driven selective sweeps can lead to co-evolutionary changes between mitochondrial and nuclear genomes [30]. However, this hypothesis requires confirmation through mitochondrial haplotype sequencing and comparative genomic analysis.

This hypothesized interpretation is further supported by the recovery of the female proportion in the second generation, which contrasts with the multi-generational cumulative nature of functional virginity. We propose that the first generation served as a selection mechanism that favored compatible hybrid individuals, contributing to the restoration of the sex ratio to normal levels.

We propose that the reproductive incompatibility in *T. remus* likely stems from a mismatch between the mitochondrial genes and the nuclear genes in infected and uninfected strains, although direct molecular evidence is needed to confirm this mechanism. In PI-*Wolbachia* systems where infected females reproduce parthenogenetically over many generations, both the mtDNA and nuclear genome may become co-adapted to the presence of the symbiont. When *Wolbachia* is removed, and sexual reproduction resumes, crosses between cured and naturally uninfected strains may generate offspring with incompatible mitochondrial-nuclear combinations. This scenario is analogous to the mitonuclear discordance observed in *Polytremis nascens*, where *Wolbachia*-associated mtDNA introgression created mismatches between mitochondrial and nuclear phylogenies [29]. This explanation does not entirely align with any of the three hypotheses mentioned earlier. The female proportion recovered in the second generation, indicating that partial incompatibility was overcome through the selection of compatible individuals. The total number of offspring and the number of female individuals in the second generation were significantly lower than those in the self-crossing group. This decline may be due to the elimination of partially incompatible individuals in the first generation or to mild reproductive suppression that persisted in the second-generation females. The recovery of traits in the second generation shows that partial incompatibility was effectively addressed through the strategic selection of compatible individuals.

(3) Effects of Symbiont-Induced PI Mechanisms

Fricke and Lindsey [5] identified two factors, named PifA and PifB, that induce parthenogenesis in PI-*Wolbachia*-infected *Trichogramma pretiosum* through genomic analyses. These proteins possess features typical of *Wolbachia*’s host-manipulating proteins and are linked to reproductive manipulation. PifA exhibits structural similarity to the Transformer protein [31], suggesting a potential role in disrupting host RNA splicing. Their research found that *Wolbachia* infection affects sex-specific splicing patterns of the tra and dsx genes in *Tr. pretiosum*, with increased expression of pifA and pifB noted during host development, particularly in adult females. This suggests that PifA and PifB may contribute to PI-type parthenogenesis by altering host sex-determination pathways. Notably, the pif genes are located within a degraded eukaryotic association module (EAM), a genomic region that also harbors cytoplasmic incompatibility and male-killing genes in other *Wolbachia* strains [5]. Despite this degraded genomic context, pifA and pifB remain functional, suggesting these effector genes are maintained due to their role in host manipulation. The Transformer-like domain in PifA shows high conservation between the two PI-*Wolbachia* strains examined (wTpre and wLcla), while other regions of PifA contain repetitive sequences that may facilitate evolutionary diversification among PI-*Wolbachia* strains [5].

Based on these insights, the reproductive barriers observed in hybrid groups in this study (e.g., abnormal sex ratios or reproductive failure in offspring) are likely related to *Wolbachia*-induced incompatibilities in host sex-determination pathways, rather than the obligate PI observed in *Asobara japonica* or *Leptopilina clavipes* [32,33].

### 4.1. Limitations of Antibiotic Treatment

Whether residual low-titer *Wolbachia* can still influence host reproduction remains unclear in current research. The antibiotic treatment protocol used in this study followed established methods that have been widely applied in previous studies [7,8]. Although we cannot completely exclude the possibility that trace amounts of *Wolbachia* may have persisted in the Wcure strain, several lines of evidence suggest that any residual infection did not substantially affect our results. First, the Wcure♂ × W- ♀ cross showed significant reproductive incompatibility, and the reciprocal cross (W- ♂ × Wcure♀) yielded similar results, indicating that the observed reproductive barriers are consistent regardless of the direction of the cross. This consistency strongly suggests that even if residual *Wolbachia* were present, they had negligible effects on the reproductive incompatibility observed. Furthermore, Figure 1 and Table 1 clearly demonstrate the loss of parthenogenesis-inducing ability following *Wolbachia* removal, indicating that even if low-titer *Wolbachia* remained, they were insufficient to induce PI.

Regarding potential off-target effects of prolonged antibiotic exposure, the Wcure strain in this study was treated with tetracycline for five generations, followed by five generations of antibiotic-free rearing until *Wolbachia* was undetectable for five consecutive generations. Importantly, in the Wcure♂ × W- ♀ cross, the W- females had never been exposed to antibiotics, but the results are similar to the W- ♂ × Wcure♀ cross. This indicates that the observed reproductive incompatibility is not attributable to direct antibiotic exposure effects.

### 4.2. Future Directions

Several avenues remain for future investigation to further elucidate the mechanisms underlying the reproductive incompatibility observed in this study. First, quantitative PCR (qPCR) could be employed to quantify *Wolbachia* titers, which would serve two purposes: (1) confirming complete elimination of *Wolbachia* in the Wcure strain, and (2) determining the threshold titer required to induce parthenogenesis. Second, mitochondrial haplotype sequencing and single-nucleotide polymorphism (SNP) analysis could be conducted to characterize genetic differences between strains and to determine whether the observed reproductive incompatibility has a genetic basis. Third, mating behavior observations could be performed to assess whether reproductive incompatibility results from behavioral differences between strains. The latter two approaches would help determine whether the reproductive barrier occurs pre-zygotically (behavioral isolation) or post-zygotically (genetic incompatibility), providing deeper insights into the nature and timing of the reproductive isolation observed in *T. remus*.

## 5. Conclusions

This study confirms that partial reproductive incompatibility exists among *T. remus* strains after *w*Rem removal. This isolation differs from the previously proposed “functional virginity” and is more consistent with putative *Wolbachia*-induced mitonuclear incompatibility. The partial recovery of sex ratios in the second-generation hybrids suggests that this incompatibility is subject to selection and can be partially reversible. However, the total number of offspring and female counts remain lower than in self-crosses, indicating mild residual reproductive suppression.

The mechanisms of PI-*Wolbachia* are highly complex, involving multiple independent evolutionary events and producing diverse phenotypic effects on the host. Although the specific role of PI-*Wolbachia* in the reproductive isolation of *T. remus* remains to be fully elucidated, infected strains have demonstrated superior reproductive advantages in mass rearing, suggesting that their potential as biological control agents warrants further investigation. However, given the reproductive impacts that PI-*Wolbachia* (*w*Rem) may induce, future studies must carefully assess the safety of utilizing infected strains in biocontrol programs. Furthermore, developing strategies to prevent symbiont loss and mitigate the spread of *Wolbachia*-associated traits within strains will be essential for practical applications and effective population management.

## Figures and Tables

**Table 1:** Descriptive statistics of offspring production and estimated male proportions among different crossing treatments in *Telenomus remus*.

Treatment	*n*	Total Offspring (Mean ± sd)	Female Offspring (Mean ± sd)	Male Offspring (Mean ± sd)	Male Ratio (Mean)	Male Proportion (SE)	95% CI	Group
W+♀	36	45.06 ± 27.97	42.72 ± 27.08	2.58 ± 2.01	0.072	0.057 (0.022)	0.026–0.120	a
Wcure♀	206	37.19 ± 31.48	2.40 ± 10.29	34.79 ± 27.79	0.946	0.935 (0.011)	0.910–0.954	e
W-♂* W-♀	247	41.25 ± 27.02	32.38 ± 22.67	8.95 ± 7.60	0.232	0.217 (0.016)	0.187–0.249	bc
Wcure♂* Wcure♀	239	43.33 ± 28.18	31.59 ± 25.64	11.64 ± 14.50	0.313	0.269 (0.017)	0.237–0.304	c
W-♂* Wcure♀	240	46.80 ± 34.69	2.20 ± 8.24	44.59 ± 34.75	0.943	0.953 (0.008)	0.935–0.966	e
Wcure♂* W-♀	224	46.29 ± 29.93	6.58 ± 17.10	39.71 ± 30.72	0.874	0.858 (0.013)	0.830–0.882	d
W-♂* Wcure/W-♀	192	21.96 ± 23.94	18.83 ± 20.63	3.15 ± 3.91	0.150	0.143 (0.021)	0.107–0.190	ab
Wcure♂* Wcure/W-♀	189	35.56 ± 31.59	29.72 ± 27.0	5.51 ± 6.25	0.143	0.156 (0.017)	0.125–0.193	ab

Notes: *n* = number of replicates (individual females tested per treatment); sd = standard deviation; SE = standard error; 95% CI = 95% asymptotic confidence interval. Male ratio (mean) represents the raw arithmetic mean of the male proportion calculated from observed data. Male proportion (SE) represents the estimated probability from quasi-binomial generalized linear models (GLMs), with standard error in parentheses. Different letters in the “Group” column indicate statistically significant differences (*p* < 0.05, Tukey’s HSD post hoc test). “/” denotes hybrid offspring (e.g., W-/Wcure indicates F1 hybrids derived from crosses between W- and Wcure strains). “*” denotes a cross between two strains (e.g., W-♂ *W-♀ indicates a cross between a W- male and a W- female). Strain abbreviations: W+ = *Wolbachia*-infected strain; W- = naturally *Wolbachia*-uninfected strain; Wcure = *Wolbachia*-cured strain.

## Data Availability

The original contributions presented in this study are included in the article/Supplementary Materials. Further inquiries can be directed to the corresponding author.

## Supplementary Materials

The following supporting information can be downloaded at: https://www.mdpi.com/article/10.3390/biology15030210/s1, Figure S1: Agarose gel electrophoresis results confirming the absence of *Wolbachia* infection in the cured strain (Wcure) over five consecutive generations; Table S1: Total number of offspring among different treatments; Table S2: Post hoc pairwise comparisons of total offspring number among treatments; Table S3: Post hoc pairwise comparisons of female offspring number among treatments; Table S4: Post hoc pairwise comparisons of the male ratio among treatments.
