# Supplementary material for: Curing Parthenogenesis-Inducing (PI) Wolbachia-Induced Reproductive Disorders in the Egg Parasitoid Telenomus remus
Source: Biology (Basel). 2026 Jan 23;15(3):210. doi: 10.3390/biology15030210 (PMC12896621; doi:10.3390/biology15030210)
Supplement: Supplementary file 1 [file biology-15-00210-s001.zip › biology-4107729-supplementary.pdf]

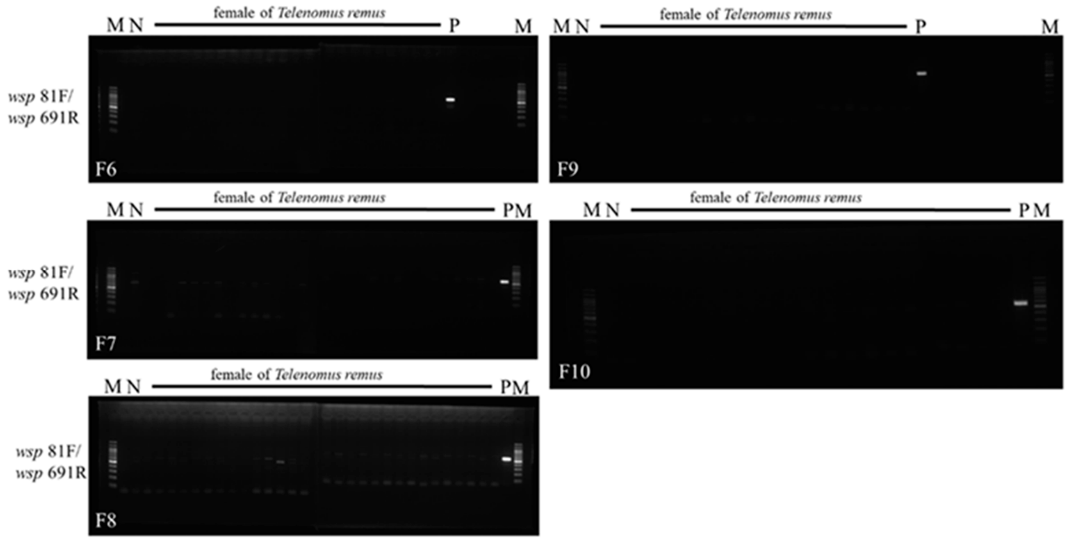

Figure S1. Electrophoresis analysis of *Wolbachia* infection in female *Telenomus remus* from the PI-*Wolbachia*-removed strain across five consecutive generations (F6–F10) using primers *wsp* 81F/*wsp* 691R. M: 100 bp DNA ladder; N: negative control (ddH_2O); P: positive control.

Table S1. Total number of offspring among different treatments. Values represent estimated response with standard error (SE), degrees of freedom (df), and 95% asymp-totic confidence intervals (asymp.LCL–asymp.UCL). Different letters in the group column indicate significant differences ($p < 0.05$)

Treatment	response	SE	df	asymp. LCL	asymp. UCL	.group
W+♀	45.06	6.912	Inf	33.36	60.86	bc
Wcure♀	37.19	2.392	Inf	32.79	42.19	bc
W-♂ * W-♀	41.25	2.419	Inf	36.77	46.27	bc
Wcure♂ * Wcure♀	43.33	2.581	Inf	38.55	48.69	bc
W-♂ * Wcure♀	46.80	2.779	Inf	41.66	52.58	c
Wcure♂ * W-♀	46.29	2.845	Inf	41.03	52.21	bc
W-♂ * Wcure/W-♀	21.96	1.479	Inf	19.25	25.06	a
Wcure♂ * Wcure/W-♀	35.56	2.389	Inf	31.17	40.56	b

Notes: "/" denotes hybrid offspring (e.g., Wcure/W- indicates F1 hybrids derived from crosses between Wcure and W- strains).

Table S2. Post-hoc pairwise comparisons of total offspring number among treatments. Values represent ratios with standard error (SE), degrees of freedom (df), z-ratio, and *p*-values. Comparisons with $p < 0.05$ indicate significant differences between treatment pairs

contrast	ratio	SE	df	null	z.ratio	*p*.value
(W- ♂ * W- ♀) / (W- ♂ * Wcure/W- ♀)	1.878	0.1677	Inf	1	7.059	< 0.001
(W- ♂ * W- ♀) / (W- ♂ * Wcure ♀)	0.8814	0.07356	Inf	1	-1.512	0.801
(W- ♂ * W- ♀) / (W+ ♀)	0.9156	0.1504	Inf	1	-0.5372	≈ 1.0
(W- ♂ * W- ♀) / Wcure ♀	1.109	0.09652	Inf	1	1.190	0.935
(W- ♂ * W- ♀) / (Wcure ♂ * W- ♀)	0.8912	0.07571	Inf	1	-1.356	0.877
(W- ♂ * W- ♀) / (Wcure ♂ * Wcure/W- ♀)	1.160	0.1035	Inf	1	1.666	0.709
(W- ♂ * W- ♀) / (Wcure ♂ * Wcure ♀)	0.9521	0.07958	Inf	1	-0.5873	≈ 1.0
(W- ♂ * Wcure/W- ♀) / (W- ♂ * Wcure ♀)	0.4693	0.04213	Inf	1	-8.426	< 0.001
(W- ♂ * Wcure/W- ♀) / (W+ ♀)	0.4875	0.08167	Inf	1	-4.289	< 0.001
(W- ♂ * Wcure/W- ♀) / Wcure ♀	0.5905	0.05498	Inf	1	-5.658	< 0.001
(W- ♂ * Wcure/W- ♀) / (Wcure ♂ * W- ♀)	0.4745	0.04327	Inf	1	-8.176	< 0.001
(W- ♂ * Wcure/W- ♀) / (Wcure ♂ * Wcure/W- ♀)	0.6177	0.05876	Inf	1	-5.064	< 0.001
(W- ♂ * Wcure/W- ♀) / (Wcure ♂ * Wcure ♀)	0.5069	0.04557	Inf	1	-7.557	< 0.001
(W- ♂ * Wcure ♀) / (W+ ♀)	1.039	0.1709	Inf	1	0.2309	≈ 1.0
(W- ♂ * Wcure ♀) / Wcure ♀	1.258	0.1101	Inf	1	2.625	0.147
(W- ♂ * Wcure ♀) / (Wcure ♂ * W- ♀)	1.011	0.08642	Inf	1	0.1293	≈ 1.0
(W- ♂ * Wcure ♀) / (Wcure ♂ *Wcure/W- ♀)	1.316	0.1180	Inf	1	3.065	0.0453
(W- ♂ * Wcure ♀) / (Wcure ♂ * Wcure ♀)	1.080	0.09085	Inf	1	0.9169	0.985
(W+ ♀) / Wcure ♀	1.211	0.2015	Inf	1	1.153	0.945
(W+ ♀) / (Wcure ♂ * W- ♀)	0.9734	0.1609	Inf	1	-0.1630	≈ 1.0
(W+ ♀) / (Wcure ♂ * Wcure/W- ♀)	1.267	0.2122	Inf	1	1.414	0.851
(W+ ♀) / (Wcure ♂ * Wcure ♀)	1.040	0.1711	Inf	1	0.2378	≈ 1.0
Wcure ♀ / (Wcure ♂ * W- ♀)	0.8036	0.07149	Inf	1	-2.458	0.214
Wcure ♀ / (Wcure ♂ * Wcure/W- ♀)	1.046	0.09729	Inf	1	0.4845	≈ 1.0
Wcure ♀ / (Wcure ♂ * Wcure ♀)	0.8585	0.07525	Inf	1	-1.741	0.660
(Wcure ♂ * W- ♀) / (Wcure ♂ * Wcure/W- ♀)	1.302	0.1185	Inf	1	2.896	0.0733
(Wcure ♂ * W- ♀) / (Wcure ♂ * Wcure ♀)	1.068	0.09145	Inf	1	0.7719	0.995
(Wcure ♂ * Wcure/W- ♀) / (Wcure ♂ * Wcure ♀)	0.8206	0.07368	Inf	1	-2.201	0.351

Notes: *p*-values of 1.00 indicate non-significant differences, where treatment pairs showed nearly identical mean values (ratio ≈ 1.0). "/" denotes hybrid offspring (e.g., Wcure/W- indicates F1 hybrids derived from crosses between Wcure and W- strains).

Table S3. Post-hoc pairwise comparisons of female offspring number among treatments. Values represent ratios with standard error (SE), degrees of freedom (df), z-ratio, and *p*-values. Comparisons with $p < 0.05$ indicate significant differences between treatment pairs

contrast	ratio	SE	df	null	z.ratio	*p*.value
(W- ♂ * W- ♀) / (W- ♂ * Wcure/W- ♀)	1.720	0.2861	Inf	1	3.260	0.0247
(W- ♂ * W- ♀) / (W- ♂ * Wcure ♀)	14.72	2.384	Inf	1	16.61	< 0.001
(W- ♂ * W- ♀) / (W+ ♀)	0.7580	0.2331	Inf	1	-0.9010	0.986
(W- ♂ * W- ♀) / Wcure ♀	13.48	2.270	Inf	1	15.44	< 0.001
(W- ♂ * W- ♀) / (Wcure ♂ * W- ♀)	4.925	0.7923	Inf	1	9.910	< 0.001
(W- ♂ * W- ♀) / (Wcure ♂ * Wcure/W- ♀)	1.089	0.1817	Inf	1	0.5138	≈ 1.0
(W- ♂ * W- ♀) / (Wcure ♂ * Wcure ♀)	1.025	0.1605	Inf	1	0.1587	≈ 1.0
(W- ♂ * Wcure/W- ♀) / (W- ♂ * Wcure ♀)	8.558	1.477	Inf	1	12.44	< 0.001
(W- ♂ * Wcure/W- ♀) / (W+ ♀)	0.4407	0.1380	Inf	1	-2.616	0.150
(W- ♂ * Wcure/W- ♀) / Wcure ♀	7.836	1.400	Inf	1	11.52	< 0.001
(W- ♂ * Wcure/W- ♀) / (Wcure ♂ * W- ♀)	2.863	0.4914	Inf	1	6.130	< 0.001
(W- ♂ * Wcure/W- ♀) / (Wcure ♂ * Wcure/W- ♀)	0.6334	0.1122	Inf	1	-2.578	0.164
(W- ♂ * Wcure/W- ♀) / (Wcure ♂ * Wcure ♀)	0.5960	0.09987	Inf	1	-3.088	0.0422
(W- ♂ * Wcure ♀) / (W+ ♀)	0.05150	0.01601	Inf	1	-9.541	< 0.001
(W- ♂ * Wcure ♀) / Wcure ♀	0.9156	0.1599	Inf	1	-0.5053	≈ 1.0
(W- ♂ * Wcure ♀) / (Wcure ♂ * W- ♀)	0.3346	0.05598	Inf	1	-6.544	< 0.001
(W- ♂ * Wcure ♀) / (Wcure ♂ *Wcure/W- ♀)	0.07401	0.01281	Inf	1	-15.05	< 0.001
(W- ♂ * Wcure ♀) / (Wcure ♂ * Wcure ♀)	0.06964	0.01136	Inf	1	-16.33	< 0.001
(W+ ♀) / Wcure ♀	17.78	5.589	Inf	1	9.156	< 0.001
(W+ ♀) / (Wcure ♂ * W- ♀)	6.497	2.016	Inf	1	6.030	< 0.001
(W+ ♀) / (Wcure ♂ * Wcure/W- ♀)	1.437	0.4505	Inf	1	1.157	0.944
(W+ ♀) / (Wcure ♂ * Wcure ♀)	1.352	0.4167	Inf	1	0.9797	0.977
Wcure ♀ / (Wcure ♂ * W- ♀)	0.3654	0.06345	Inf	1	-5.798	< 0.001
Wcure ♀ / (Wcure ♂ * Wcure/W- ♀)	0.08084	0.01448	Inf	1	-14.04	< 0.001
Wcure ♀ / (Wcure ♂ * Wcure ♀)	0.07607	0.01290	Inf	1	-15.19	< 0.001
(Wcure ♂ * W- ♀) / (Wcure ♂ * Wcure/W- ♀)	0.2212	0.03806	Inf	1	-8.769	< 0.001
(Wcure ♂ * W- ♀) / (Wcure ♂ * Wcure ♀)	0.2082	0.03375	Inf	1	-9.679	< 0.001
(Wcure ♂ * Wcure/W- ♀) / (Wcure ♂ * Wcure ♀)	0.9410	0.1581	Inf	1	-0.3622	≈ 1.0

Notes: *p*-values of 1.00 indicate non-significant differences, where treatment pairs showed nearly identical mean values (ratio ≈ 1.0)."/" denotes hybrid offspring (e.g., Wcure/W- indicates F1 hybrids derived from crosses between Wcure and W- strains).

Table S4. Post-hoc pairwise comparisons of the male ratio among treatments. Val-ues represent odds ratios with standard error (SE), degrees of freedom (df), z-ratio, and p-values. Comparisons with p < 0.05 indicate significant differences between treatment pairs

contrast	odds.ratio	SE	df	null	z.ratio	*p*.value
(W- ♂ * W- ♀) / (W- ♂ * Wcure/W- ♀)	1.654	0.3231	Inf	1	2.574	0.165
(W- ♂ * W- ♀) / (W- ♂ * Wcure ♀)	0.01363	0.002693	Inf	1	-21.75	< 0.001
(W- ♂ * W- ♀) / (W+ ♀)	4.569	1.951	Inf	1	3.559	0.0089
(W- ♂ * W- ♀) / Wcure ♀	0.01908	0.003894	Inf	1	-19.40	< 0.001
(W- ♂ * W- ♀) / (Wcure ♂ * W- ♀)	0.04575	0.006601	Inf	1	-21.38	< 0.001
(W- ♂ * W- ♀) / (Wcure ♂ * Wcure/W- ♀)	1.491	0.2409	Inf	1	2.472	0.207
(W- ♂ * W- ♀) / (Wcure ♂ * Wcure ♀)	0.7495	0.09559	Inf	1	-2.261	0.316
(W- ♂ * Wcure/W- ♀) / (W- ♂ * Wcure ♀)	0.008244	0.002013	Inf	1	-19.65	< 0.001
(W- ♂ * Wcure/W- ♀) / (W+ ♀)	2.763	1.244	Inf	1	2.257	0.318
(W- ♂ * Wcure/W- ♀) / Wcure ♀	0.01154	0.002879	Inf	1	-17.88	< 0.001
(W- ♂ * Wcure/W- ♀) / (Wcure ♂ * W- ♀)	0.02767	0.005631	Inf	1	-17.63	< 0.001
(W- ♂ * Wcure/W- ♀) / (Wcure ♂ * Wcure/W- ♀)	0.9017	0.1949	Inf	1	-0.4788	< 0.001
(W- ♂ * Wcure/W- ♀) / (Wcure ♂ * Wcure ♀)	0.4533	0.08704	Inf	1	-4.121	< 0.001
(W- ♂ * Wcure ♀) / (W+ ♀)	335.2	151.3	Inf	1	12.88	< 0.001
(W- ♂ * Wcure ♀) / Wcure ♀	1.400	0.3516	Inf	1	1.339	0.884
(W- ♂ * Wcure ♀) / (Wcure ♂ * W- ♀)	3.356	0.6899	Inf	1	5.889	< 0.001
(W- ♂ * Wcure ♀) / (Wcure ♂ *Wcure/W- ♀)	109.4	23.85	Inf	1	21.53	< 0.001
(W- ♂ * Wcure ♀) / (Wcure ♂ * Wcure ♀)	54.98	10.68	Inf	1	20.63	< 0.001
(W+ ♀) / Wcure ♀	0.004176	0.001897	Inf	1	-12.06	< 0.001
(W+ ♀) / (Wcure ♂ * W- ♀)	0.01001	0.004312	Inf	1	-10.69	< 0.001
(W+ ♀) / (Wcure ♂ * Wcure/W- ♀)	0.3263	0.1425	Inf	1	-2.564	0.169
(W+ ♀) / (Wcure ♂ * Wcure ♀)	0.1640	0.06978	Inf	1	-4.250	< 0.001
Wcure ♀ / (Wcure ♂ * W- ♀)	2.397	0.5079	Inf	1	4.127	< 0.001
Wcure ♀ / (Wcure ♂ * Wcure/W- ♀)	78.14	17.50	Inf	1	19.46	< 0.001
Wcure ♀ / (Wcure ♂ * Wcure ♀)	39.28	7.889	Inf	1	18.28	< 0.001
(Wcure ♂ * W- ♀) / (Wcure ♂ * Wcure/W- ♀)	32.59	5.584	Inf	1	20.33	< 0.001
(Wcure ♂ * W- ♀) / (Wcure ♂ * Wcure ♀)	16.38	2.289	Inf	1	20.02	< 0.001
(Wcure ♂ * Wcure/W- ♀) / (Wcure ♂ * Wcure ♀)	0.5027	0.07917	Inf	1	-4.367	< 0.001

Notes: "/" denotes hybrid offspring (e.g., Wcure/W- indicates F1 hybrids derived from crosses between Wcure and W- strains).
